# From learners to teachers: a peer-assisted learning model in undergraduate orthopedic education: a quasi-experimental study

**DOI:** 10.25122/jml-2026-0031

**Published:** 2026-03

**Authors:** Ionut Dudau, Dumitru Sutoi, Bogdan Chiu, Daian Ionel Popa, Raluca Radbea, George Marin, Anda Nicoleta Ciontos, Vlad Mulcutan-Chis, Dragos Fortofoiu, Maria Sutoi, Ovidiu Alexandru Mederle, Bogdan Nicolae Deleanu

**Affiliations:** 1 Doctoral School, Faculty of General Medicine, Victor Babes University of Medicine and Pharmacy Timisoara, Timisoara, Romania; 2 Department of Surgery, Emergency Discipline, Victor Babes University of Medicine and Pharmacy, Timisoara, Romania; 3 Emergency Municipal Clinical Hospital, Timisoara, Romania; 4 Victor Babes University of Medicine and Pharmacy, Timisoara, Romania; 5 Department of Cardiology, Institute of Cardiovascular Diseases, Timisoara, Romania; 6 Doctoral School, University of Medicine and Pharmacy of Craiova, Craiova, Romania; 7 Pius Brinzeu Emergency County Hospital, Timisoara, Romania

**Keywords:** peer-assisted learning, medical students, orthopedic education, undergraduate medical education, self-confidence

## Abstract

Contemporary medical education emphasizes student-centered approaches that better align with modern learners. Peer-assisted learning (PAL) has proven effective in enhancing knowledge, clinical skills, engagement, and professional development. Although widely integrated into Western European curricula, its implementation remains limited in countries such as Romania. This study aimed to evaluate PAL within undergraduate orthopedic training, focusing on its benefits for both learners and peer instructors, as well as its potential to inform teaching practices. A total of 50 medical students participated in the study. Significant improvements were observed across all assessed domains following the intervention, including increased confidence in procedural skills such as suturing, wound debridement, and cast application, as well as greater understanding of osteosynthesis principles (*P* < 0.05). Anxiety-related measures significantly decreased, including fear of performing procedures and making technical mistakes. Perceptions of PAL also improved, with higher levels of trust in peer tutors and comfort in peer-led learning environments. No significant gender-based differences were identified. PAL was associated with significant improvements in students’ perceived confidence across key orthopedic procedural skills and a marked reduction in anxiety related to clinical performance. The intervention promoted a supportive learning environment that facilitated active participation, reduced hierarchical barriers, and increased trust in peer tutors. These findings are consistent with existing literature supporting student-centered and experiential learning approaches in medical education. Overall, PAL proved to be an effective and feasible educational strategy, enhancing both technical competence and psychological readiness. Its scalability and high acceptability highlight its potential for broader integration into undergraduate medical curricula.

## Introduction

In the contemporary educational context, an increasingly pronounced discrepancy can be observed between traditional pedagogical paradigms and the learning profile of the modern student. Although foundational, conventional teaching methods often prove insufficient in meeting the current imperative to develop transversal competencies. Consequently, methodological restructuring becomes essential, moving beyond mere information transmission to prioritize the cultivation of critical thinking, problem-solving capacity, and effective interpersonal communication. The limitations of traditional, teacher-centered approaches and the need to shift toward active, student-centered learning strategies have been consistently emphasized in the literature [1,2].

In this context, peer-assisted learning (PAL) has emerged as an effective educational strategy, demonstrating significant improvements in knowledge acquisition, practical skills, and learner satisfaction compared to traditional teaching methods [3], while also contributing to increased motivation, self-efficacy, and engagement among both learners and peer tutors [4]. PAL represents a valuable educational strategy in professional training, exerting a significant impact on the consolidation of practical competencies. Through horizontal interaction, this model facilitates the effective transition from theoretical understanding to clinical or technical application, while providing a supportive learning environment that reduces performance-related anxiety [5]. Consequently, PAL enhances learners’ confidence in performing complex procedures and fosters a rigorous collaborative climate essential for the development of communication and teamwork skills aligned with contemporary academic and professional standards [6].

Engagement in PAL, particularly through peer teaching roles, enhances students’ self-efficacy, communication, collaboration, and leadership skills. It also promotes a shift toward a more patient-centered and professionally oriented learning approach, contributing significantly to the development of clinical competence and professional identity [7]. Peer teaching is increasingly used in medical education because it engages students actively as both learners and educators. Evidence indicates that students taught by peers perform similarly to those instructed by faculty, particularly in problem-based learning and clinical skills training. Additionally, acting as a peer teacher enhances the tutor’s own understanding of the subject matter. Despite initial concerns regarding teaching confidence, structured training can address these limitations. Overall, peer teaching offers a mutually beneficial approach that supports both knowledge acquisition and student engagement [8].

PAL has demonstrated its effectiveness over the last few decades and is widely discussed in the medical field. This method is based on the social reciprocity theory: students learn better when they interact with peers who share similar social roles and knowledge levels [9]. The advantages of PAL are both for the student who teaches (tutor) and the student who learns (tutee). Studies found that the psychological stress of asking questions and talking to an expert or professor is reduced in PAL as the tutor is also a student. As for the subject itself, the tutor’s recent understanding of the materials makes it easier to explain difficult concepts. More than that, this teaching method is highly effective compared to other hands-on teaching methods for gaining practical or clinical skills. The most interesting fact regarding this method is that it forces the tutor to consolidate deep knowledge by filling cognitive gaps and increasing tutors’ confidence in their professional capabilities [10,11].

In the broader context of medical education, the adoption of innovative, student-centered teaching strategies such as PAL and reverse teaching is particularly relevant for countries like Romania, where traditional pedagogical models still predominate. The integration of such approaches has the potential to facilitate alignment with contemporary European educational standards by promoting active learning, critical thinking, and the development of transversal competencies. Therefore, implementing and systematically evaluating these methods may represent an important step toward modernizing medical education and enhancing the overall quality of training in line with Western European practices. This need is further supported by evidence indicating that medical education systems in countries such as Romania still face challenges in adopting innovative, student-centered teaching strategies and digital learning approaches, underscoring the need for continuous modernization and alignment with international standards [12,13]. In contrast, several Western European countries have already successfully integrated PAL into their medical curricula, demonstrating its effectiveness and adaptability across diverse educational settings. In the United Kingdom, PAL is widely used to complement traditional teaching and address gaps in formal training, particularly in the development of teaching competencies. Evidence from UK-based programs indicates that PAL can deliver clinical skills training with outcomes comparable to faculty-led instruction, while simultaneously enhancing students’ confidence, teaching abilities, and professional development [14]. Similarly, in Germany, PAL has become a well-established component of medical education, particularly within skills labs, where student tutors play an active role in teaching practical skills. National data show that PAL programs are implemented in nearly all medical faculties, reflecting their strong curricular integration despite structural variations [15]. In the Netherlands, PAL is actively incorporated into clinical training, especially to support the development of clinical reasoning during undergraduate clerkships. Structured approaches, such as paired consultations, have been shown to promote collaborative learning, psychological safety, and active peer engagement, emphasizing the importance of supportive educational environments in maximizing the effectiveness of PAL [16].

Taken together, these international experiences highlight the potential of PAL as a scalable and effective educational strategy, reinforcing the need for its broader implementation and evaluation in medical education systems where such approaches are not yet fully integrated.

The primary objective of this study was to evaluate PAL as an educational strategy within undergraduate orthopedic training, with a focus on its benefits for both learners and peer instructors. Furthermore, the study aimed to explore the potential of PAL to inform and inspire faculty members to integrate similar student-centered approaches into their teaching practices, based on the educational outcomes achieved.

## Material and Methods

### Study design and setting

This quasi-experimental study was conducted in December 2025 at the Faculty of Medicine of the Victor Babes University of Medicine and Pharmacy in Timisoara. The study evaluated the effectiveness of a PAL educational intervention implemented during a practical orthopedic workshop.

### Participants (eligibility criteria and recruitment)

At the beginning of December 2025, 20 fourth-, fifth-, and sixth-year medical students who had completed the orthopedic curriculum and demonstrated a sustained interest in the specialty were invited to participate as student teachers Interest was evidenced through direct discussions with the study supervisor, attending the Orthopedics ward beyond required university hours, completing summer practice in the Orthopedics clinic, or volunteering within the department. Of these, 10 students agreed to participate, while the remainder either declined or were unavailable.

Eligibility criteria for trainers included completion of the fourth-year orthopedic module and expressed interest in orthopedics. All participating trainers provided written informed consent in accordance with the General Data Protection Regulation (GDPR). For workshop participants, a registration form was distributed over 7 days via WhatsApp and student study groups to medical students enrolled at the Faculty of Medicine within the university. Inclusion criteria were enrollment as a general medicine student and completion of the registration form among the first 50 respondents. No prioritization criteria were applied beyond the order of registration.

Exclusion criteria included refusal to participate, incomplete or incorrectly completed registration forms, and lack of eligibility (e.g., non–general medicine students). A total of 64 registration forms were submitted; 3 were excluded due to ineligibility or incomplete data, and 11 were excluded due to late submission after capacity was reached.

### Intervention (educational procedure)

Following enrollment, the 10 selected student teachers participated in a structured 4-hour training session delivered by orthopedic physicians. The training mirrored the format of the subsequent practical workshop. It focused on five core orthopedic procedures: surgical knot tying, suturing, cast application for long bone fracture immobilization, wound debridement, and plate-and-screw osteosynthesis. An additional instructional component was included during the training session: a “tips and tricks” module delivered by the facilitators that covered teaching strategies and the educational objectives expected to be achieved during the workshop. Participants completed a pre-test before the training and a post-test immediately after, as described below.

### Workshop implementation (PAL model)

The practical workshop employing the PAL model was structured into 10 stations. Each student teacher was assigned to a group of five participants. Before the workshop, all participants completed a pre-test identical to the one administered to the trainers 1 week earlier (30 minutes).

The workshop consisted of two main components:

Demonstration phase (90 minutes): Five orthopedic procedures were presented, each by pairs of student teachers, with 15 minutes allocated to each procedure.

Hands-on practice phase (120 minutes): Participants practiced all five procedures under the supervision and guidance of the student teachers, receiving individualized feedback.

At the conclusion of the workshop, a structured Q&A session was conducted.

### Data collection and outcome measures

Data were collected using pre- and post-intervention tests administered to both trainers and participants. Each test was completed within 30 minutes. The structure and content of the assessment tools are detailed in the corresponding subsection. The primary outcome measure was the difference between pre-test and post-test scores, reflecting knowledge and skill acquisition following the PAL intervention.

### Ethical considerations

All participants provided informed consent before inclusion in the study. Data was collected and processed anonymously, in compliance with GDPR. Participation in the study did not expose individuals to any identifiable risk.

### Form development and structure

Data was collected using two structured, study-specific assessment instruments: one designed for workshop participants (learners) and one for student teachers. Both instruments were administered in a pre-test/post-test format to evaluate the impact of the educational intervention. The instrument for workshop participants consisted of knowledge-based multiple-choice questions (MCQs) assessing core orthopedic procedural understanding related to the five targeted skills (knot tying, suturing, cast application, wound debridement, and plate-and-screw osteosynthesis). These items were designed to evaluate the understanding of procedural steps, indications, and correct technical execution. In addition, the questionnaire included self-assessment items measured on Likert-type scales, capturing participants’ perceived confidence, perceived usefulness of the training, and attitudes toward PAL.

The trainer instrument followed a similar structure, incorporating both objective knowledge-based items (MCQs) and perception-based Likert-scale items, allowing for evaluation of both cognitive gains and educational perceptions.

### Rationale for developing an original instrument

An original assessment tool was developed due to the lack of validated instruments specifically designed to measure short-term knowledge acquisition and changes in perception following procedural, skills-based orthopedic training delivered through a peer-assisted learning model. Existing instruments in medical education are typically focused on long-term competency assessment or general educational satisfaction and therefore do not adequately capture immediate procedural learning outcomes combined with attitudinal shifts toward PAL. The development of a customized instrument enabled precise alignment among the intervention’s learning objectives, the specific orthopedic procedures taught, and the evaluation of both cognitive and perceptual outcomes.

### Content development and validity

The questionnaire items were developed by the study authors in collaboration with orthopedic specialists involved in the training process. The initial draft of the questionnaire was evaluated by a panel of three orthopedic specialists. They reviewed the items in the Forms separately to assess clinical accuracy and relevance to the targeted procedures. All items were directly derived from the instructional content delivered during the training and workshop. Content validity was established through expert review, with orthopedic physicians assessing the clinical accuracy of the items, their relevance to the targeted procedures, and their alignment with the intended educational objectives.

### Data analysis

Statistical analysis was conducted using Microsoft Excel (2024) and JASP (version 0.95.4). Continuous variables were reported as medians and interquartile ranges (IQRs), based on data normality assessed by the Shapiro–Wilk test. Skewness and Kurtosis were presented to describe the distribution compared to the normal one. Values of *P* < 0.05 were categorized as statistically significant (rejecting the null hypothesis). The Wilcoxon signed-rank test was used to evaluate differences between pre- and post-intervention results for non-normally distributed data, while the Mann–Whitney U test was used to determine differences between two groups (in this case, gender). The questionnaire’s internal consistency was assessed using Cronbach’s Alpha, with values above 0.7 considered acceptable.

## Results

The final database consisted of 50 medical students who participated in the medical workshop described. Of the total, 26 (52%) were women, and 24 (48%) were men. The median age was 23, with an interquartile range of 21-25 years. To evaluate the internal consistency of the questionnaire, Cronbach’s alpha was calculated for all pre-intervention categories, revealing values of 0.940, 0.916, and 0.936 (clinical confidence and skill readiness, clinical anxiety and psychological safety, and perception of peer-assisted learning). Secondly, the final database used to analyze the student teachers comprised 10 distinct students.

As shown in Table 1, all parameters analyzed showed statistically significant differences between the pre- and post-course assessments (*P* < 0.05, Wilcoxon signed-rank test). The largest difference between pre- and post-course assessments was observed in students’ perceptions of their ability to irrigate and debride a wound, understanding the biomechanics of plate and screws, and their confidence to apply a cast or splint. All of these parameters present a rise in the median from 5 to 9, a four-point median increase between pre- and post-course evaluation.

**Table 1:** Comparison of pre- and post-intervention perception of students

Category		Median (IQR)	Skewness	Kurtosis	*P* value
I feel confident in my ability to correctly identify and use standard surgical instruments (e.g., needle drivers, forceps).	Before	6 (3–9)	-0.220	-1.031	<0.001*
	After	8 (7–9)	-0.431	0.057	
I feel confident in my ability to properly irrigate and debride a contaminated wound.	Before	5 (1–9)	-0.128	-1.048	<0.001*
	After	9 (7–10)	-0.722	-0.157	
I feel confident in my ability to place and tie simple interrupted sutures independently.	Before	4.5 (1–9.25)	-0.045	-1.440	<0.001*
	After	8 (2–10)	-1.064	2.023	
I understand the underlying biomechanical principles of how osteosynthesis plates provide stability.	Before	5 (2–8)	0.011	-0.987	<0.001*
	After	9 (8–10)	-0.742	-0.122	
I feel confident in my ability to apply a cast or splint while maintaining the limb’s correct anatomical position.	Before	5 (1.25–8.75)	-0.225	-1.177	<0.001*
	After	9 (8–10)	-0.924	0.970	
Overall, I feel well-prepared for the practical, hands-on requirements of my upcoming clinical rotations.	Before	6 (2–10)	-0.198	-1.125	<0.001*
	After	9 (8–10)	-1.121	1.482	
I feel anxious about performing basic orthopedic or surgical maneuvers on a real patient.	Before	8 (6–10)	-0.813	0.396	<0.001*
	After	4 (1.25–6.75)	0.383	-0.211	
I am concerned about making technical mistakes when practicing new medical procedures.	Before	7 (5.25–8.75)	-0.699	0.121	<0.001*
	After	4 (1–7)	0.505	-0.237	
I feel intimidated when asking questions about practical skills in a traditional, professor-led environment.	Before	7.5 (4.75–10)	-0.374	-0.800	<0.001*
	After	4 (1.25–6.75)	0.246	-0.806	
I feel overwhelmed by the amount of physical coordination required to perform surgical and orthopedic knots.	Before	7 (5–9)	-0.685	-0.210	<0.001*
	After	4 (1–7)	0.190	-0.478	
I believe that learning practical medical skills from a near-peer (fellow student) is an effective educational method.	Before	7 (4–10)	-0.773	-0.018	<0.001*
	After	9 (8–10)	-0.418	-0.659	
I feel comfortable being observed and corrected by my peers during a practical simulation.	Before	6 (3.25–8.75)	-0.654	-0.018	<0.001*
	After	9 (9–9)	-1.197	1.701	
I trust that my top-performing peers have the theoretical knowledge required to teach me effectively.	Before	7 (4.25–9.75)	-0.516	-0.346	<0.001*
	After	9 (7–10)	-0.374	-1.017	
I feel more comfortable asking “basic” or “silly” questions to a fellow student than I do to a senior faculty member.	Before	7 (4–10)	-0.388	-0.339	<0.001*
	After	9 (7–10)	-1.155	1.140	
I believe that having a peer explain a concept helps bridge the gap between textbook theory and practical application.	Before	7 (5–9)	-0.811	0.153	<0.001*
	After	9 (7–10)	-0.351	-0.885	

* Statistically significant, P <0.05, Wilcoxon signed-rank test

**Figure 1:**
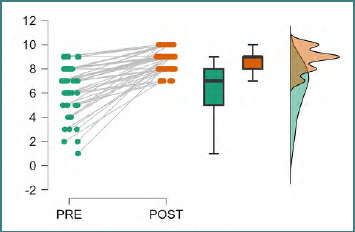
Learners’ perception of PAL efficiency (Wilcoxon signed-rank test)

The general perception about PAL’s efficiency before and after the intervention indicated a consistent upward trend across the cohort. As shown in the raincloud plot, all students reported a perceived improvement in PAL as an efficient teaching method (Figure 1). From an initial median of 7 with an interquartile range of 4–10, we can see that after the workshop, the median rose to 9 with an IQR of 8–10.

**Figure 2:**
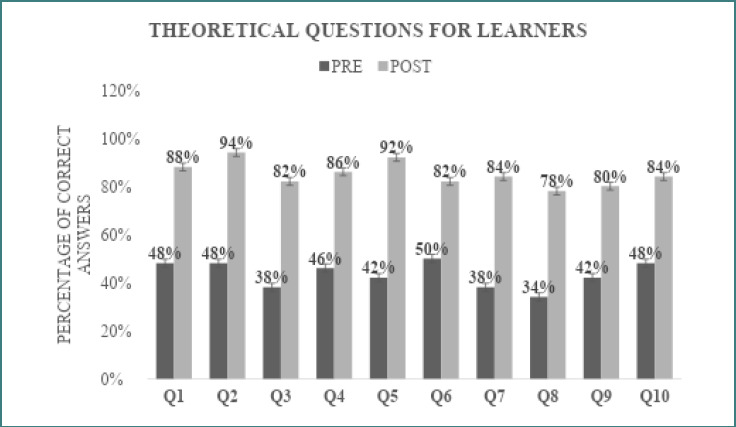
Evolution of theoretical knowledge

The gain in theoretical knowledge before and after the intervention is illustrated in Figure 2. A significant improvement was observed across all ten questions (Supplementary File), indicating an overall increase in theoretical understanding. At baseline, the lowest level of knowledge was related to the application of fiberglass plaster for the treatment of long bone fractures, with only 34% of students answering correctly. Following the workshop, this proportion increased to 78%. The greatest improvement was observed in the question regarding the correct positioning of the needle holder at the start of suturing, where correct responses increased from 42% pre-intervention to 92% post-intervention.

Supplementary File

A gender-based pairwise comparison was conducted using the Mann–Whitney U test to assess whether perceptions of PAL differed between male and female participants. As shown in Table 2, no statistically significant difference was observed between genders for any of the variables analyzed. In almost all areas observed, both men and women reported a similar level of anxiety, confidence, and surgical skill perception. Following the intervention, both groups demonstrated improvements across all analyzed areas, with similar growth patterns. The high IQR variance, especially before the workshop, indicated a high degree of individuality based on personal experience, which narrowed significantly after the intervention. Finally, the absence of statistically significant differences between groups suggests that PAL is similarly accessible and well perceived across genders.

**Table 2:** Pairwise comparison between genders

Variable		Median (IQR) ales	Median (IQR) Females	*P* value
I feel confident in my ability to correctly identify and use standard surgical instruments (e.g., needle drivers, forceps).	Before	5 (1–10)	6 (3-9)	0.399
	After	8 (6–10)	8 (7-9)	0.527
I feel confident in my ability to properly irrigate and debride a contaminated wound.	Before	4.5 (1–8.5)	5 (2-8)	0.260
	After	9 (7–10)	9 (7.25-10)	0.968
I feel confident in my ability to place and tie simple interrupted sutures independently.	Before	4 (1–9)	5 (1-9)	0.837
	After	8.5 (6.5–10)	8 (6.25-9.75)	0.216
I understand the underlying biomechanical principles of how osteosynthesis plates provide stability.	Before	5.5 (1.5–9.5)	5 (2.25-7.75)	0.914
	After	9 (7.75–10)	9 (7.25-10)	0.936
I feel confident in my ability to apply a cast or splint while maintaining the limb’s correct anatomical position.	Before	5 (1–9)	6.5 (3.5-9.5)	0.522
	After	9 (8–10)	9 (8-10)	0.648
Overall, I feel well-prepared for the practical, hands-on requirements of my upcoming clinical rotations.	Before	6 (1–10)	6 (3-9)	0.867
	After	9 (8–10)	9 (8-10)	0.807
I feel anxious about performing basic orthopedic or surgical maneuvers on a real patient.	Before	8 (6–10)	7.5 (5.5-9.5)	0.368
	After	4 (2.75)	3 (1-6)	0.430
I am concerned about making technical mistakes when practicing new medical procedures.	Before	8 (6.5–9.5)	7 (5.25-8.75)	0.533
	After	4 (1–7)	3 (1-5.75)	0.953
I feel intimidated when asking questions about practical skills in a traditional, professor-led environment.	Before	8 (5.5–10)	7 (4.25-9.75)	0.875
	After	5 (1–9)	4 (2-6)	0.409
I feel overwhelmed by the amount of physical coordination required to perform surgical and orthopedic knots.	Before	7.5 (5–10)	7 (5-9)	0.867
	After	4 (1–7)	3.5 (1-6.5)	0.805
I believe that learning practical medical skills from a near-peer (fellow student) is an effective educational method.	Before	7 (4–10)	7 (4-10)	0.906
	After	9 (7–10)	9 (8-10)	0.431
I feel comfortable being observed and corrected by my peers during a practical simulation.	Before	7 (4–10)	6 (5-7)	0.686
	After	9 (8.75–9.25)	9 (8.25-9.75)	0.407
I trust that my top-performing peers have the theoretical knowledge required to teach me effectively.	Before	7 (4–10)	7 (5-9)	0.914
	After	9 (7–10)	9 (7.25-10)	0.259
I feel more comfortable asking “basic” or “silly” questions to a fellow student than I do to a senior faculty member.	Before	7 (3–10)	6.5 (3.5-9.5)	0.421
	After	9 (7–10)	9 (8-10)	0.445
I believe that having a peer explain a concept helps bridge the gap between textbook theory and practical application.	Before	7 (4.75–9.25)	7 (5-9)	0.984
	After	9 (7.75–10)	9 (7-10)	0.549

Mann–Whitney U test, with the grouping variable set to “Gender”

**Figure 3:**
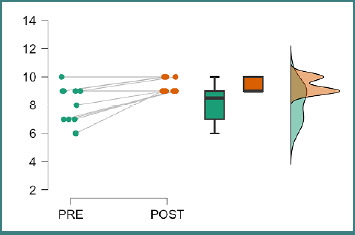
Perception about PAL efficiency for student teachers

From the student teacher’s perspective, as indicated in Figure 3, improved perceptions of PAL’s efficiency were observed among almost all students who presented the workshop. All of them had a stable or positive trajectory in their perception of PAL’s efficiency as an educational tool. The post-test analysis shows a substantial upward shift in the median, with a “ceiling effect,” as seen in the density plot, where all student teacher values were between 9 and 10.

Before the workshop, only 3 of the 10 students selected to teach displayed a desire to pursue an academic career, scoring 10 on the Likert scale. Still, all students showed a strong desire to do so, with all indicating at least an 8 out of 10. After the workshop, the majority (60%) indicated they want an academic career, scoring 10 out of 10. Using this workshop, student teachers felt they could integrate theory and practice more effectively, with initial median scores of 8 and 9 after the workshop. They felt more capable of developing teaching materials for their peers. Initially, none of them scored 10 on the Likert scale, and after the workshop, two changed their answers to 10.

## Discussion

The present study demonstrates that implementing a PAL model in undergraduate orthopedic education leads to significant improvements in perceived competence and psychological readiness for clinical practice. These findings support the growing body of evidence advocating for student-centered educational strategies in medical training, particularly in procedural disciplines where experiential learning is essential [17–19]. One of the most important findings of this study is the consistent and statistically significant increase in self-reported confidence across all assessed procedural domains, including suturing, wound debridement, cast application, and understanding of osteosynthesis principles. These results are in line with previous research demonstrating that PAL enhances procedural confidence and self-efficacy, which are critical predictors of clinical performance [20–22]. The magnitude of improvement observed in our cohort may be explained by the combination of cognitive congruence and immediate hands-on practice, both of which have been identified as key mechanisms underlying the effectiveness of peer teaching [23,24].

A particularly relevant finding is the substantial reduction in anxiety-related variables, including fear of performing procedures on real patients, fear of making technical mistakes, and intimidation in traditional faculty-led environments. This aligns with the theoretical framework of social congruence, which suggests that peer interactions reduce hierarchical barriers and create a psychologically safe learning environment [25,26]. Previous studies have shown that reduced anxiety not only improves learner satisfaction but also enhances skill acquisition and retention, particularly in simulation-based training [27,28]. Conversely, some authors argue that reduced perceived pressure in peer-led environments may lead to underexposure to real clinical stressors, highlighting the need for a balanced integration with faculty-led teaching [29].

Moreover, our results demonstrate a significant increase in the perceived effectiveness and acceptability of PAL, including greater trust in peer tutors and greater comfort with being observed and corrected by peers. This is consistent with findings from systematic reviews indicating high levels of student satisfaction and perceived educational value associated with PAL interventions [30–32]. The increased comfort in asking “basic” questions further supports previous observations that peer learning environments facilitate active participation and reduce fear of negative evaluation [33,34].

Interestingly, no significant gender-based differences were identified in either baseline or post-intervention outcomes, suggesting that the benefits of PAL are broadly applicable across diverse student populations. Similar findings have been reported in previous studies, indicating that PAL is an inclusive educational strategy that does not disproportionately benefit or disadvantage specific demographic groups [35,36]. However, some literature suggests that gender-related differences may emerge in larger cohorts or different cultural contexts, warranting further investigation [36]. Within the context of Romanian medical education, where traditional pedagogical models remain prevalent, these findings are particularly relevant. The results demonstrate that even a short, structured PAL intervention can yield measurable educational benefits, supporting its feasibility and scalability in settings undergoing curricular modernization [37,38]. Comparative data from Western European institutions further reinforce PAL’s adaptability across different educational systems, highlighting its potential as a universal teaching strategy [39,40].

From an educational standpoint, the study also highlights the role of PAL in facilitating the integration of theoretical knowledge with practical application, as reflected by increased agreement that peer explanations help bridge this gap. The knowledge gains regarding plaster cast application (increased from 34% to 78%) and needle holder positioning (increased from 42% to 92%) highlight the aspects that medical students understand least. An exact reason for which these procedures are perceived as the most complicated theoretical standpoint, but it can orient further studies and orthopedic medical education to tackle this finding. This finding is supported by cognitive learning theories, which emphasize the importance of contextualized learning and near-peer explanation in enhancing knowledge transfer. Additionally, the reciprocal nature of PAL has been shown to reinforce learning for both tutors, contributing to deeper understanding and long-term retention [41,42].

Although the present study focused primarily on learners, it is important to acknowledge the educational benefits for peer tutors, including the development of teaching competencies, leadership skills, and professional identity formation. These outcomes have been consistently reported in the literature and represent an important added value of PAL interventions [43–45].

In addition, the present findings are consistent with previous research in medical education, which emphasizes the importance of innovative, student-centered learning strategies for improving engagement, confidence, and professional skill development among medical students. Studies conducted in Romanian cohorts have highlighted similar benefits of interactive, hands-on educational approaches, particularly in enhancing motivation, self-confidence, and perceived preparedness for clinical practice [46–48]. These findings further support the role of structured educational interventions in facilitating the transition from theoretical knowledge to clinical application, especially in educational systems where traditional teaching models still predominate.

### Study limitations

Several limitations should be acknowledged. First, the study was conducted in a single academic center with a relatively small sample size, which may limit generalizability. Second, reliance on self-reported measures introduces the possibility of response bias, although these measures remain relevant for assessing confidence and perceptions [49]. Third, the short-term design precludes assessment of long-term knowledge retention and skill sustainability, an aspect highlighted as a limitation in previous PAL research [50]. Additionally, the absence of a control group limits direct comparison with traditional teaching methods. While some studies report equivalent outcomes between peer-led and faculty-led instruction, others suggest that hybrid models may yield optimal results by combining the strengths of both approaches [51].

Another limitation is the absence of longitudinal follow-up, which prevents the evaluation of sustained impact over time. Previous studies in medical education have emphasized the importance of assessing long-term retention and behavioral changes following educational interventions, particularly in relation to clinical preparedness and professional development [46-48].

### Future directions

Future research should explore integrating PAL into broader educational frameworks that combine hands-on training, interdisciplinary collaboration, and digital learning strategies. Evidence from recent studies suggests that blended and experiential educational models can significantly enhance student engagement, confidence, and career orientation in medical education [47,48]. Future research should incorporate objective assessments of clinical skills, longitudinal follow-up, and randomized controlled designs to strengthen the evidence base. Expanding PAL implementation across multiple specialties and institutions would further validate its scalability and generalizability [52].

Furthermore, the development of standardized training programs for peer tutors is essential to ensure consistency and maintain educational quality, as variability in tutor preparation has been identified as a key factor influencing PAL effectiveness [53,54].

## Conclusion

This study demonstrates that PAL is an effective and feasible approach in undergraduate orthopedic education. The implementation of a structured, hands-on workshop significantly increased students’ perceived confidence in key procedural skills and reduced anxiety about clinical performance. Beyond cognitive gains, PAL fostered a supportive learning environment that encouraged active participation and reduced hierarchical barriers. The high level of acceptance and trust in peer tutors further supports its integration into medical curricula. Consistent with previous research, student-centered and experiential strategies enhance motivation, confidence, and clinical preparedness. Overall, PAL represents a scalable method for improving both technical competence and psychological readiness.

## Data Availability

The datasets generated and/or analyzed during the current study are not publicly available due to institutional and privacy considerations, but are available from the corresponding author upon reasonable request. Any requests for data access will be considered in accordance with ethical approvals and data protection regulations.
