# Supplementary material for: From learners to teachers: a peer-assisted learning model in undergraduate orthopedic education: a quasi-experimental study
Source: J Med Life. 2026 Mar;19(3):210–9. doi: 10.25122/jml-2026-0031 (PMC13155161; doi:10.25122/jml-2026-0031)
Supplement: Supplementary file 1 [file JMedLife-19-210-s001.pdf]

THEORETICAL QUESTIONS

1. What is the most universally recommended fluid for the initial, high-volume irrigation of a contaminated open fracture or acute wound?
a) 10% Povidone-Iodine (Betadine)
b) Hydrogen Peroxide
c) Chlorhexidine gluconate
d) Normal Saline (0.9% NaCl)

2. During the initial cleaning of a traumatic wound, why is it critical to aggressively debride (remove) visibly necrotic or devitalized tissue?
a) To stimulate immediate skin growth over the defect.
b) To stop capillary-level bleeding.
c) To remove dead tissue that acts as a breeding ground for bacterial infection.
d) To create enough physical space for the orthopedic hardware.

3. Which of the following suture materials is absorbable and therefore the best choice for closing deep tissue layers (like fascia or subcutaneous fat)?
a) Polypropylene (Prolene)
b) Silk
c) Polyglactin 910 (Vicryl)
d) Nylon

4. When holding a curved needle with a needle driver, where should the needle be grasped to maximize control and prevent it from bending or breaking?
a) Exactly at the sharp tip.
b) Exactly at the swage (where the thread connects to the needle).
c) Approximately one-third to one-half the distance from the swage to the tip.
d) Directly in the middle of the needle's curve.

5. When performing a standard instrument tie with a needle holder to secure a simple interrupted suture, where must the needle holder be positioned to begin the first throw?
a) Directly over the wound edge.
b) Exactly in the middle, between the two suture strands.
c) Parallel to the needle's curve.
d) Clamped onto the needle itself.

6. When removing interrupted sutures, why is it important to cut the suture right next to the skin edge and pull the knot across the wound?
a) To avoid cutting the patient's skin with the scissors.
b) To avoid pulling the contaminated, exposed portion of the suture track through the clean healing tissue underneath.
c) To save time during the removal process.
d) To ensure the knot doesn't untie.

7. When a surgeon applies a standard metal plate and screws to a fractured bone, what is the primary biomechanical goal?
a) To permanently carry the entire weight of the patient's limb.
b) To stimulate a massive inflammatory response for healing.
c) To hold the bone fragments in anatomical reduction and provide stability while the bone heals.
d) To deliver local antibiotics directly into the bone marrow.

8. When applying a plaster or fiberglass splint to conservatively treat a fractured long bone, what is the fundamental orthopedic rule regarding the length of the splint?
a) Immobilize only the specific bone that is fractured.
b) Immobilize the joint above and the joint below the fracture.
c) Always immobilize the limb from the fingertips/toes all the way to the torso.
d) Leave all joints free to prevent muscle atrophy.

© 2026 by the authors. This article is an open access article distributed under the terms and conditions of the Creative Commons Attribution (CC BY 4.0) license.

9. You just applied a cast to a patient's fractured forearm. What is the most critical early warning sign that the cast is too tight and the patient might be developing Compartment Syndrome?
a) The patient feels a mild throbbing sensation.
b) The plaster feels warm to the touch.
c) The patient experiences severe pain out of proportion to the injury, especially when you passively stretch their fingers.
d) The patient's skin becomes itchy under the cast.

10. When positioning a patient's foot and ankle for the application of a short leg cast, what is the optimal position to prevent Achilles tendon contracture and ensure normal walking mechanics later?
a) Maximum plantarflexion (pointing the toes down).
b) Maximum dorsiflexion (pulling the toes up).
c) Neutral position (approximately 90 degrees).
d) Slight inversion of the ankle.

© 2026 by the authors. This article is an open access article distributed under the terms and conditions of the Creative Commons Attribution (CC BY 4.0) license.
